# A Bipyridine-Ester Dual-Modified 2,2,6,6-Tetramethylpiperidin-1-oxyl Derivative for Aqueous Organic Redox Flow Batteries

**DOI:** 10.3390/ma18122770

**Published:** 2025-06-12

**Authors:** Qianqian Zheng, Yanwen Ren, Cuicui He, Jingjing Nie, Binyang Du

**Affiliations:** 1 State Key Laboratory (SKL) of Biobased Transportation Fuel Technology, Department of Polymer Science & Engineering, Zhejiang University, Hangzhou 310058, China; 22229025@zju.edu.cn (Q.Z.); ywren@zju.edu.cn (Y.R.); 22429001@zju.edu.cn (C.H.); 2 Department of Chemistry, Zhejiang University, Hangzhou 310058, China; niejj@zju.edu.cn

**Keywords:** energy storage, aqueous organic redox flow battery, TEMPO derivatives

## Abstract

The transition to renewable energy makes energy storage crucial. Aqueous organic redox flow batteries (AORFBs) show great potential in large-scale energy storage due to their outstanding safety compared to conventional systems. Derivatives of 2,2,6,6-tetramethylpiperidin-1-oxyl (TEMPO) show significant promise as catholyte materials in AORFBs. In this work, a bipyridine-ester dual-modified TEMPO derivative, (2,2,6,6-tetramethyl-1-piperidinyloxy)carbonyl-ethyl-(4-(pyridin-4-yl)benzyl) ammonium bromide (TEMP-BPy) was successfully synthesized via a two-step functionalization. The synthesized compound was experimentally confirmed to possess excellent electrochemical stability. The electron-withdrawing effect of the 4,4′-bipyridine moiety elevates the redox potential by 60 mV. When implemented as a catholyte paired with methyl viologen (MV) as the anolyte in AORFB, the TEMP-BPy/MV system demonstrates excellent performance: achieving a cell voltage of 1.28 V and an energy density of 14.5 Wh L^−1^ at a 0.6 M (16.08 Ah L^−1^) concentration with 71.3% material utilization. Notably, it demonstrates exceptional cycling stability with an average capacity retention of 99.86% per cycle over 200 cycles, and it exhibits particularly impressive initial stability, with an average capacity retention of 99.997% per cycle during the first 100 cycles.

## 1. Introduction

The rapid global transition toward renewable energy systems has elevated energy storage technologies as critical infrastructure, particularly in maintaining grid resilience and enabling the efficient utilization of intermittent renewable sources [1,2]. Against this backdrop, aqueous organic redox flow batteries (AORFBs) have emerged as a promising technology for large-scale energy storage due to their inherent safety, cost-effectiveness, and tunable electrochemical properties inherent to organic redox-active materials [3,4,5,6]. Unlike traditional inorganic systems such as vanadium flow batteries, which face challenges of resource scarcity and corrosive electrolytes, AORFBs utilize sustainable organic molecules that enable decoupling energy and power scalability [7,8,9].

Among the explored organic electrolytes, 2,2,6,6-tetramethylpiperidine-1-oxyl (TEMPO) derivatives stand out as particularly promising catholyte materials, leveraging their rapid single-electron transfer kinetics and exceptional hydrolytic stability to achieve unprecedented cycling performance in neutral pH environments [10,11]. However, critical limitations persist, including the instability of reduced intermediates, the crossover of active species through membranes, and insufficient energy density under neutral pH conditions [12]. To address these problems, structural modification at the 4-position has been identified as a critical pathway. Hydrophilic functional groups have been introduced, such as hydroxyl (-OH) [11], sulfonate (-SO_3_^−^) [13], carboxylate (-COO^−^) [14], quaternary ammonium (-N^+^(CH_3_)_3_) [15], and other hydrophilic structures [16]. These groups synergistically enhance the aqueous solubility of the obtained TEMPO derivatives and suppress their radical dimerization via electrostatic shielding effects [17]. However, some of the charged groups can easily trigger the ring-opening degradation of TEMPO derivatives and compromise the cycle stability of the corresponding redox flow batteries (RFBs).

Chang et al. [18] incorporated imidazole, along with an ester group, into TEMPO. The electron-withdrawing imidazolium substituent increases the redox potential of the resultant TEMPO derivatives by 0.15 V compared to that of 4-OH-TEMPO. This cationic modification enabled remarkable cycling stability of the corresponding RFBs, achieving an average capacity fade rate of merely 0.15% per cycle at 0.1 M electrolyte and 0.11% per cycle at 0.4 M electrolyte. Fan et al. [19] reported a novel TEMPO derivative featuring “π–π” conjugated imidazolium and “p–π” conjugated acetylamino groups. The synergistic conjugation effects endowed the material with unprecedented temporal stability, maintaining an average daily capacity retention of 99.95% over 16.7 days. Hu et al. [20] proposed a viologen-decorated TEMPO derivative ((TPABPy)Cl_3_) for neutral RFBs. The optimized structure delivered exceptional energy density (19.0 Wh L^−1^ at 1.5 M concentration), alongside ultra-stable cycling performance (averaged a capacity retention of 99.95% per cycle). These structural optimizations collectively reveal that π-conjugated architectures facilitate intramolecular charge transport pathways, effectively mitigating the inherent capacity degradation of TEMPO-based electrode materials [21].

In this study, we developed a bipyridine-ester dual-modified TEMPO derivative (2,2,6,6-tetramethyl-1-piperidinyloxy)carbonyl-ethyl-(4-(pyridin-4-yl)benzyl) ammonium bromide (TEMP-BPy) through a two-step synthetic route involving bromination and quaternization. The strong electron-withdrawing effect of 4,4′-bipyridine can significantly raise the redox potential. The engineered material exhibits a +60 mV anodic shift in redox potential versus that of commercial 4-OH-TEMPO, yielding a cell voltage of 1.28 V in 1.5 M NaCl electrolyte. Furthermore, the increase in cationic charge density effectively suppresses the membrane crossover of catholyte materials via Donnan exclusion effects. When coupled with methyl viologen (MV), which is a cost-effective anolyte material with superior redox reversibility [11], the optimized AORFB exhibited exceptional cycling stability, sustaining an average capacity retention per cycle of 99.86% over 200 cycles and a particularly impressive initial stability, with an average capacity retention of 99.997% per cycle during the first 100 cycles. Additionally, the TEMP-BPy/MV AORFB delivered a record energy density of 14.5 Wh L^−1^ at a concentration of 0.6 M (16.08 Ah L^−1^) under a current density of 30 mA cm^−2^.

## 2. Materials and Methods

### 2.1. Materials

All chemicals used in this study were procured from commercial suppliers and employed without further purification: 4-hydroxy-2,2,6,6-tetramethylpiperidin-1-oxyl (4-OH-TEMPO, Aladdin Chemical Ltd., Shanghai, China), 4-bromobutyryl chloride (Aladdin), chloroacetic acid (Macklin Biochemical Co., Shanghai, China), and 1,3-propanesultone (Macklin). 4,4′-bipyridine (J&K Chemical Ltd., Shanghai, China) was stored in a desiccator. Cation-exchange membranes (Selemion AMVN) were procured from Asahi Glass Co., Tokyo, Japan. Graphite felt electrodes (3 mm thickness, Jingu Carbon Material Co., Shenyang, China) were thermally activated at 400 °C for 6 h in air, while porous carbon electrodes (Jingtan Technology Co., Langfang, China) were polished with sandpaper before use.

### 2.2. Synthesis of TEMP-BPy

**Scheme 1:**
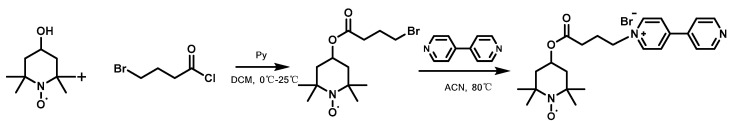
The synthetic route of TEMP-BPy.

The synthetic route of the target product—(2,2,6,6-tetramethyl-1-piperidinyloxy)carbonyl-ethyl-(4-(pyridin-4-yl)benzyl) ammonium bromide (TEMP-BPy)—is shown in Scheme 1. In the first step, the intermediate (2,2,6,6-tetramethyl-1-piperidinyloxy)carbonyl-3-bromopropyl (TEMP-Br) was synthesized via a nucleophilic substitution reaction. In a 500 mL three-necked flask equipped with a stirrer, 4-OH-TEMPO (196 mmol, 33.76 g) and pyridine (216 mmol, 17.06 g) were sequentially added to dichloromethane (DCM) in an ice bath; then, 4-bromobutyryl chloride (216 mmol, 40.06 g) was added dropwise under stirring. The reaction mixture was stirred for 24 h at room temperature. After the reaction, the white precipitate was removed by filtration, and the filtrate was extracted with deionized water, 10% NaHCO_3_ aqueous solution, 2% HCl aqueous solution, and finally deionized water three times. The resulting organic solution was dried over anhydrous Na_2_SO_4_ overnight and concentrated under vacuum to obtain a red viscous liquid (yield: 90%). In the second step, 4,4′-bipyridine was introduced via a quaternization reaction. A mixture of TEMP-Br (32.12 g, 100 mmol) from the previous step and 4,4′-bipyridine (18.72 g, 120 mmol) was dissolved in 300 mL of acetonitrile (ACN) solution. The reactants were heated to 75 °C and refluxed for 24 h. The product was precipitated with acetone three times and concentrated under vacuum at 40 °C. Finally, a tan solid, TEMP-BPy, was obtained (yield: 87%).

### 2.3. Material Characterization

^1^H NMR spectra were acquired on a Bruker AVANCE III 400 MHz spectrometer (Bruker Corp., Billerica, MA, USA). Electron paramagnetic resonance (EPR) measurements were conducted on a Bruker A300 spectrometer (Bruker Corp., MA, USA). ESI-MS data were collected with a LCMS-2020 spectrometer (Shimadzu, Kyoto, Japan) equipped with an ESI source. FT-IR spectra were recorded with a Bruker TENSOR II spectrometer (Bruker Corp., MA, USA). UV-Vis spectra were measured using an Agilent Cary 300 spectrometer (Varian Australia Pty Ltd., Victoria, Australia). Physicochemical properties were determined using the Anton Paar (Graz, Austria) instrumentation: density measurements were carried out with a DMA 4100 digital density meter (Anton Paar Graz, Graz, Austria), and viscosities were measured using an AMVn automated microviscometer (Anton Paar Graz, Austria). 

### 2.4. Electrochemical Characterization

Cyclic voltammetry (CV) measurements were carried out using a standard three-electrode setup. This setup consisted of a glassy carbon working electrode, a platinum wire counter electrode, and a Ag/AgCl reference electrode. The voltage sweep range was set from 0.2 V to 0.9 V, while the scan rate was systematically varied from 20 mV s^−1^ to 500 mV s^−1^. Rotating disk electrode (RDE) measurements coupled with linear sweep voltammetry (LSV) were also performed via a three-electrode configuration, which contained a glassy carbon rotating disk as the working electrode, a graphite rod as the counter electrode, and a Ag/AgCl reference electrode. At a fixed scan rate of 10 mV s^−1^, the rotating rate of the working electrode was gradually changed from 300 rpm to 2400 rpm. The variation in the rotating rate enabled a detailed study of the mass-transfer effects on the electrochemical processes. The Levich equation was used to determine the diffusion coefficient:

(1) $$I_{L}=0.62nFAD^{2/3}v^{-1/6}w^{1/2}C$$

where $I_{L}$ is the limiting diffusion current density, n is the number of reacting electrons, F is Faraday’s constant (F = 96485 C mol^−1^), A is the electrode area (A = 0.196 cm^2^), D is the diffusion coefficient, v is the kinetic viscosity of the fluid (v = 0.011 cm^2^ s^−1^), w is the electrode rotational rate, and C is the concentration of the active substance (C = 1 mM).

The kinetic current (*I_k_*) was extracted from Koutecký–Levich plots of reciprocal current density versus reciprocal square root of rotation rate (*ω*^1/2^) at selected constant overpotentials. C is the concentration of the active substance (C = 1 mM). Subsequently, the charge-transfer rate constant (*k*_0_) was quantified using the Butler–Volmer equation:

(2) $$I_{0}=nFAK_{0}C$$

where $I_{0}$ is the current density at the equilibrium potential when the rates of oxidation and reduction reactions are equal.

### 2.5. Flow Battery Tests

A laboratory-scale flow battery was assembled with two graphite felt electrodes (2 cm × 2 cm × 3 mm), a Selemion AMVN anion-exchange membrane (Asahi Glass Co., Tokyo, Japan), and two graphite plates as current collectors. Additionally, the electrolyte circulation system consisted of two peristaltic pumps (BT100-1L, Longer Precision Pump Co., Baoding, China) operating at a flow rate of 20 mL min^−1^. Galvanostatic cycling tests were performed in the voltage range of 0.3 to 1.75 V using a Neware BTS CT-4008Tn-5V6A-S1 battery test system (Neware Co., Shenzhen, China) under ambient conditions.

## 3. Results and Discussion

### 3.1. Chemical Characterization of TEMP-BPy

#### 3.1.1. Structure and Composition of TEMP-BPy

TEMP-BPy was synthesized through two processes. As illustrated in Scheme 1, 4-OH-TEMPO was first esterified with 4-bromobutyryl chloride, followed by combination with 4,4′-bipyridine via a quaternization reaction. Each step was purified through filtration or precipitation, achieving an impressive overall yield exceeding 78%. This facile synthesis approach facilitates industrial large-scale production. Furthermore, the synthesis of TEMP-BPy demonstrates significantly lower material costs compared to those of commercially available VRFBs (Table S1). The structural and compositional analysis of the synthesized product was investigated by ^1^H-NMR and ESI-MS. Note that TEMP-BPy was reduced by ascorbic acid prior to ^1^H NMR characterization in order to avoid radical effects. The ^1^H NMR spectrum (Figure S1) of reduced TEMP-BPy clearly shows the ^1^H signals of the 4,4′-bipyridine moieties at δ 7.85, 8.34, 8.66, and 8.88 ppm, along with distinct methyl group signals at δ 1.31 ppm from the TEMPO component. However, during the quaternization reaction, a small portion of the bipolar structure TEMP-BPy-TEMP is inevitably formed, as illustrated in Scheme S2. Given that this by-product shares similar physicochemical properties with the target main product, TEMP-BPy, separating the two has been proven to be extremely challenging. The efforts to remove TEMP-BPy-TEMP finally failed, despite trying various purification methods, including column chromatography. Based on the ^1^H NMR integration results, the percentage of TEMP-BPy-TEMP can be estimated to be about 15%. Nevertheless, TEMP-BPy-TEMP does not have a pronounced negative impact on the redox activity of the cathodic structure on the TEMPO side. Therefore, the obtained TEMP-BPy can be utilized in the subsequent analysis. ESI-MS analysis (Figure S2a) demonstrates a molecular ion [M + H]^+^ at *m*/*z* 397.2361, which aligns precisely with the calculated value for TEMP-BPy (C_23_H_31_N_3_O_3_^+·^, *m*/*z* 397.2360), further corroborating the successful synthesis. What is more, from the full ESI-MS spectrum, TEMP-BPy-TEMP was detected at an *m*/*z* value of 638.4054 (Figure S2b). And the ratios obtained are approximately consistent with those of the ^1^H NMR analyses. The EPR spectrum exhibits a characteristic triplet signal (Figure S3), which obviously suggests the existence of stable TEMPO radicals [22,23], confirming the preservation of the paramagnetic nitroxide functionality in the final compound. The existence of the bipyridine structure was further confirmed by FT-IR spectroscopy analysis. The characteristic absorption peaks at 1641 cm^−1^ correspond to the stretching vibrations of -C=N- groups. The broad peak at 3423 cm^−1^ is attributed to the formation of bipyridinium ions after bipyridine quaternization and the interaction between the quaternary ammonium salt and water (Figure S4). Additionally, the absorption peak at 818 cm^−1^ arises from the out-of-plane bending vibration mode of the bipyridine ring.

#### 3.1.2. Solubility and Viscosity of TEMP-BPy

**Figure 1:**
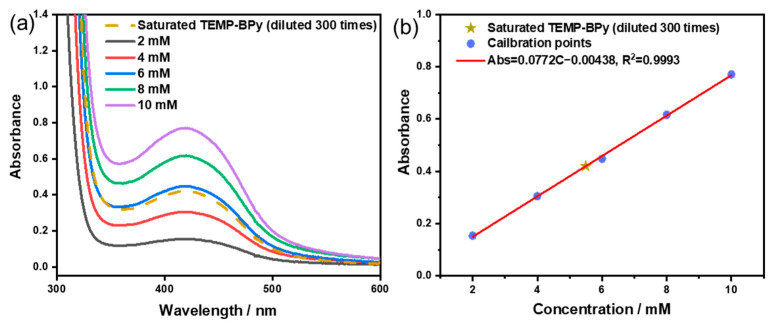
UV-Vis spectroscopic analysis for determining the maximum solubility of TEMP-BPy. (**a**) Concentration-dependent UV-Vis spectra recorded in a water solution showing a characteristic absorption band at 428 nm. (**b**) Corresponding absorbance–concentration calibration curve established from (**a**).

The aqueous solubility of TEMP-BPy was quantitatively analyzed via UV-Vis spectroscopy. Due to the n-π* transitions of the nitroxyl radical (-N-O**^·^**), there was an obvious absorbance at a wavelength of λ = 428 nm [19]. As shown in Figure 1, a series of solutions with concentrations ranging from 2 mM to 10 mM were prepared to establish a standard calibration curve. Subsequently, the saturated solution was diluted 300 times to ensure that its absorbance fell within the appropriate range. Then, the corresponding concentration could be calculated based on the standard calibration curve. According to the test results, the maximum solubility is 1.78 M in water solution, corresponding to a capacity of 47.7 Ah L^−1^. Note that the solubility value represents the total concentration of the TEMPO functional groups (including those in TEMP-BPy and TEMP-BPy-TEMP), as quantified by UV-Vis spectroscopy.

The viscosity of the electrolyte plays a crucial role in determining both system efficiency and charge carrier mobility [24]. The TEMP-BPy aqueous solution exhibits low viscosity at 20 °C, as indicated in Table S2. The viscosity of a 0.6 M TEMP-BPy solution (in 1.5 M NaCl aqueous solution) was measured to be about 2.59 mPa s, which is only a slight increase when compared to that of pure water (1.00 mPa·s at 25 °C) [13]. This minimal viscosity variation suggests that the TEMP-BPy additive does not significantly compromise the fluid’s transport properties while maintaining optimal ionic conductivity.

### 3.2. Electrochemical Performance

**Figure 2:**
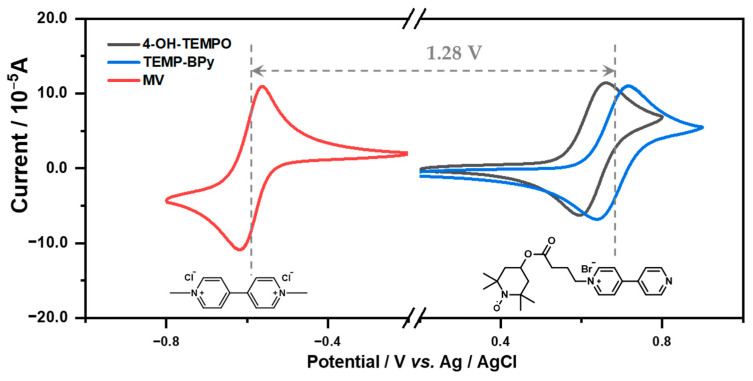
CV plots of 1 mM 4-OH-TEMPO, TEMP-BPy, and MV in a 1.5 M NaCl aqueous solution at a scan rate of 50 mV s^−1^.

The electrochemical characteristics of TEMP-BPy were initially examined by cyclic voltammetry (CV) at a concentration of 1.0 mM in 1.5 M NaCl aqueous solution. As depicted in Figure 2, the compound exhibits a well-defined oxidation peak potential at 0.72 V vs. Ag/AgCl and a reduction peak at 0.65 V vs. Ag/AgCl (blue curve), yielding a half-wave potential (E_1/2_) of 0.69 V vs. Ag/AgCl for TEMP-BPy. And it represents a 60 mV anodic shift relative to its parent compound of 4-OH-TEMPO at 0.63 V vs. Ag/AgCl (black curve). This significant potential modulation arises from the introduction of a π-conjugated 4,4′-bipyridine. The extended conjugation system facilitates intramolecular electron delocalization and effectively lowers the activation energy barrier for the redox transition [18,19,25]. Notably, for the TEMP-BPy cathode moiety, the CV plots exhibited minimal peak potential shift across scanning rates from 20 to 500 mV s^−1^, suggesting fast electron transfer kinetics (Figure S5a (right part)). Quantitative analysis using the Randles–Ševčík equation revealed excellent linear correlation (R^2^ > 0.99) between peak current and square root of scan rate (ω^1/2^), confirming diffusion-controlled redox processes and high electrochemical reversibility [26,27] (Figure S5c).

Extensive research has demonstrated that doubly substituted 4,4′-bipyridine derivatives, such as viologens, exhibit exceptional electrochemical reversibility during reduction–oxidation cycles [25,28]. However, when the 4,4′-bipyridine moiety was incorporated into the TEMP-BPy structure, forming a monosubstituted 4,4′-bipyridine structure, CV analysis revealed poor redox reversibility of the bipyridine moieties (Figure S5a (left part)). This disparity arises from the fact that the monosubstituted configuration of 4,4′-bipyridine modifies the electronic delocalization across the molecule, leading to a significant disparity in the stability between the oxidized and reduced states. Consequently, this imbalance impedes the efficient reversal of the redox process of the monosubstituted 4,4′-bipyridine structure [29,30]. Furthermore, it demonstrated an obvious deviation from the expected linear proportionality between the peak current and the square root of the scan rate (Figure S5b), proving its limited electrochemical reversibility.

Based on the electrochemical performance analysis, while these structural limitations preclude TEMP-BPy’s application in bipolar molecular configurations, the material demonstrates potential applicability as a cathodic component in AORFB systems. And for the anodic counterpart, methyl viologen (MV, 1,1′-dimethyl-4,4′-bipyridinium dichloride) was selected based on its well-established electrochemical behavior in AORFB applications [11,24,31]. MV was synthesized via a Menschutkin reaction between 4,4′-bipyridine and chloroacetic acid [32] (Scheme S1) and confirmed by ^1^H NMR (Figure S6). The MV^2+^/MV^+·^ redox couple demonstrates a characteristic half-wave potential (E_1/2_) at −0.59 V vs. Ag/AgCl (Red curve). When MV is paired with TEMP-BPy, this electrochemical combination yields a theoretical cell voltage of 1.28 V. What is more, CV studies at varying scan rates revealed that the TEMP-BPy cathode exhibits superior redox reversibility compared to the 4-OH-TEMPO, whereas MV demonstrates enhanced electrochemical reversibility relative to the TEMP-BPy anode part (Figure S7). Consequently, the TEMP-BPy/MV AORFB system was selected for subsequent investigation.

**Figure 3:**
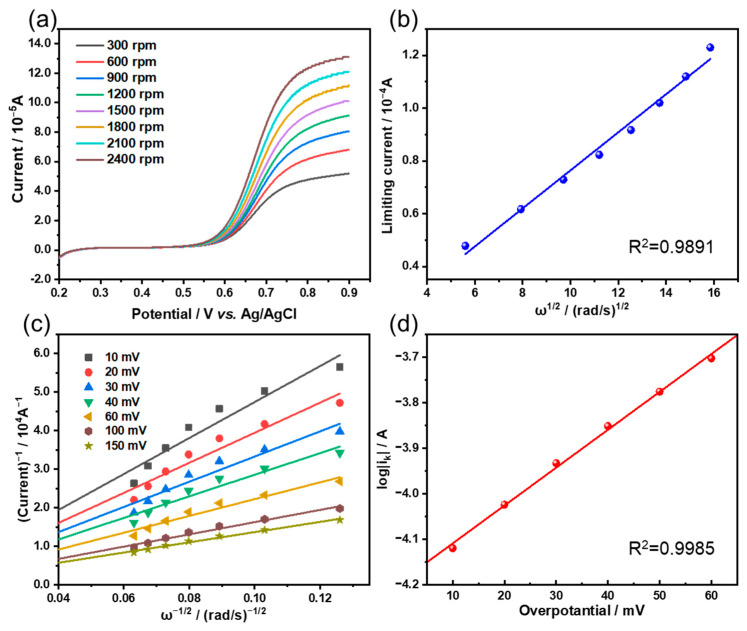
RDE tests of TEMP-BPy. (**a**) Linear sweep voltammograms of TEMP-BPy at a scan rate of 10 mV s^−1^. (**b**) Levich plots of limiting current versus the square root of rotation rates. (**c**) Koutecký–Levich plots were generated for different overpotentials. (**d**) Tafel plots were utilized to accurately determine the *k*_0_.

To further clarify the quasi-reversible and determine the kinetics of the TEMP-BPy redox process, we performed RDE with the LSV test system [33]. Measurements were conducted in a 1.5 M NaCl aqueous solution containing 5.0 mM TEMP-BPy, with rotation rates systematically varied from 300 to 2400 rpm (Figure 3). As depicted in Figure 3a, the limiting current ($I_{L}$) of all rotation rates exhibited linear dependence on the square root of the rotating rate ($w^{1/2}$) [20] (Figure 3b), consistent with convective-diffusion-controlled kinetics as described by the Levich equation. An analysis of the Levich slopes yielded a diffusion coefficient of D = 4.96 × 10^−6^ cm^2^ s^−1^, higher than values reported for conventional vanadium-based electrolytes, indicating superior mass transport properties [8]. Subsequently, kinetic analysis via the Koutecký–Levich method (Figure 3c) and Butler–Volmer equation with Tafel plots (Figure 3d) gave an electron-transfer rate constant of $k_{0}$ = 2.94 × 10^−4^ cm s^−1^.

### 3.3. Flow Battery Performance

**Figure 4:**
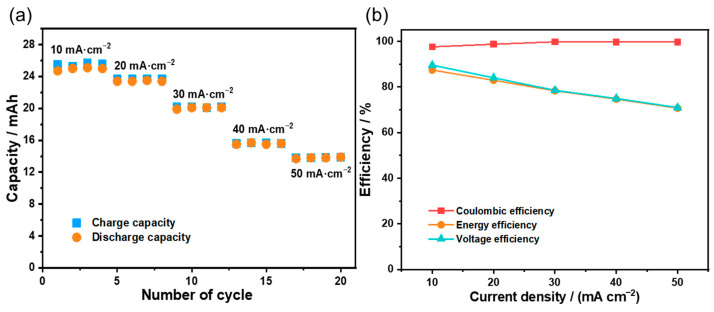
Rate capability analysis of the TEMP-BPy/MV AORFB system. (**a**) Charging and discharging capacity and (**b**) efficiency metrics (Coulombic/energy/voltage) under varied current density conditions. (Each reservoir contained 10 mL of 0.1 M TEMP-BPy and 15 mL of 0.1 M MV in a 1.5 M NaCl aqueous solution, with a theoretical storage capacity of 26.8 mAh).

To systematically assess the rate capability of the TEMP-BPy/MV AORFB system, we performed galvanostatic charge–discharge cycling experiments across a range of current densities (10–50 mA cm^−2^) in a pumped flow battery with a flow rate of 20 mL min^−1^. The battery was assembled in air with 10 mL of 0.1 M TEMP-BPy as the catholyte, 15 mL of 0.1 M MV as the anolyte, and 1.5 M NaCl aqueous solution as the supporting electrolyte. The catholyte-to-anolyte volume ratio was maintained at 2:3 to avoid the oxidative degradation side reactions of MV [34]. The current density was systematically varied from 10 to 50 mA cm^−2^ to assess performance characteristics. As shown in Figure 4a, the system demonstrated progressive capacity retention from 93.2% at 10 mA cm^−2^ to 51.5% at 50 mA cm^−2^, showing typical rate-dependent behavior for redox flow batteries. This performance degradation can be attributed to the increased cell overpotential, ohmic loss, and concentration polarization effects [4,12,35]. Correspondingly, energy efficiency decreased from 87.4% to 70.6% with increasing current density, while voltage efficiency showed a similar downward trend. At a current density of 30 mA cm^−2^, the battery achieved a practical capacity retention of 74.2%, and the charge–discharge process also occurred within a practical time frame. These results established 30 mA cm^−2^ as the optimal current density for further long-term cycling performance evaluations. Notably, the system maintained exceptional Coulombic efficiency (CE) approaching 99% across all tested current densities (Figure 4b), indicating remarkable electrochemical reversibility and stability of the TEMP-BPy/MV system. What is more, the operational voltage window remained stable between 0.3 and 1.75 V during the charge and discharge process, with no detectable gas evolution (oxygen, hydrogen, or bromine).

**Figure 5:**
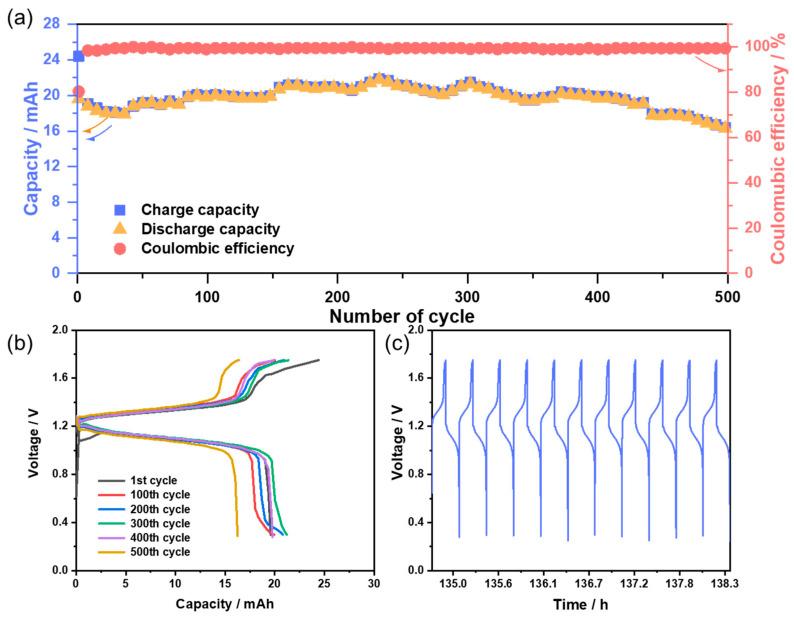
Cycling performance of the TEMP-BPy/MV AORFB system. (**a**) Galvanostatic cycling performance over 300 cycles at 30 mA cm^−2^. (**b**) Representative capacity versus time profiles. (**c**) Representative voltage versus time curves of the 300th–310th cycles. (Each reservoir contained 10 mL of 0.1 M TEMP-BPy and 15 mL of 0.1 M MV in a 1.5 M NaCl aqueous solution, with a theoretical storage capacity of 26.8 mAh).

To evaluate the long-term operational stability of the TEMP-BPy/MV AORFB system, extended galvanostatic cycling was performed at 30 mA cm^−2^ with 0.1 M active species concentrations (volume ratio of catholyte:anolyte = 2:3). As shown in Figure 5a,b, the system exhibited high cycling stability over 500 consecutive cycles. It delivered an initial discharge capacity of 19.6 mAh, corresponding to a 73.2% material utilization of its theoretical capacity (26.8 mAh). Notably, the system maintained an average capacity retention of 99.97% per cycle with a minimal fade rate of 0.03% per cycle. The Coulombic efficiency (CE) demonstrated a progressive improvement during cycling, starting at 80.1% in the initial cycle and stabilizing above 98.4% after 10 cycles. This high efficiency level persisted throughout subsequent cycling tests with an average CE of 99.2% over 500 cycles. We attribute this behavior to the oxygen-mediated passivation mechanism of MV^+·^ radicals. Residual dissolved oxygen in the electrolyte likely participates in radical scavenging reactions during the initial cycles, temporarily reducing CE [32]. Meanwhile, from the voltage–capacity curve, it can be seen that there is a minor secondary discharge plateau that emerges within the high-voltage region. This phenomenon is likely attributable to the insufficiency of the structure in the late redox stage due to the presence of certain bipolar molecules. Moreover, the AORFB exhibited well-defined charge/discharge voltage plateaus at 1.35 V (charge) and 1.09 V (discharge), respectively (Figure 5c and Figure S8), under a current density of 30 mA cm^−2^. This characteristic indicates minimal polarization and stable redox kinetics [36]. Moreover, we further investigated the CV curves of MV and TEMP-BPy after long-term cycling. As shown in Figure S9, MV and TEMP-BPy still maintain stable redox peaks, and the 50 consecutive CV scans almost overlap without offset, indicating that the active materials’ structure remains stable. The photograph of the assembled AORFB is shown in Figure S10.

To further assess the feasibility of this structure functioning as a bipolar molecule, we employed 0.1 M TEMP-BPy as both a catholyte and anolyte (volume ratio of catholyte:anolyte = 2:3). As shown in Figure S11, the system exhibited an initial charge capacity of 25.5 mAh but delivered only 2.8 mAh discharge capacity in the first cycle, corresponding to an exceptionally low Coulombic efficiency of 11%. Notably, the capacity rapidly decayed to 0.5 mAh after 80 cycles, with an average capacity retention of 98.9%. This accelerated performance degradation, characterized by irreversible redox reactions and insufficient charge recovery, directly demonstrates the structural incompatibility of TEMP-BPy as a bipolar redox-active molecule in the AORFB system.

**Figure 6:**
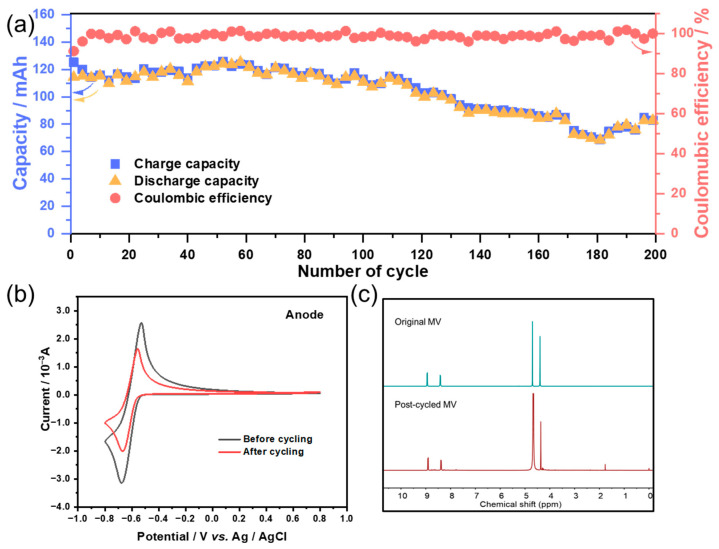
(**a**) Cycling performance of the 0.6 M TEMP-BPy/MV AORFB system. (**b**) CV tests of MV before (black) and after (red) cycling at a scan rate of 50 mV s^−1^. (**c**) ^1^H NMR spectrum of original MV (green) and post-cycled MV (red), recorded in D_2_O. (Each reservoir contained 10 mL of 0.6 M TEMP-BPy and 15 mL of 0.6 M MV in a 1.5 M NaCl aqueous solution, with a theoretical storage capacity of 160.8 mAh).

The cycling stability of the AORFB system employing 0.6 M (16.08 Ah L^−1^) TEMP-BPy/MV electrolytes was further evaluated. As shown in Figure 6a, the battery exhibited an initial discharge capacity of 114.3 mAh, corresponding to a 71.1% utilization efficiency of its theoretical capacity (160.8 mAh). It achieved an energy capacity of 14.5 Wh L^−1^, maintaining an average CE of 99.2% and an average capacity retention of 99.86% over 200 cycles. Notably, the system also exhibited exceptional average capacity retention of 99.997% with a minimal fade rate of 0.003% per cycle for the first 100 cycles, outperforming many organic redox couples in AORFB (Table S3). The observed capacity fluctuations primarily stem from thermal variations and experimental uncertainties inherent in the measurement process [31]. This remarkable stability might originate from the extended π-conjugated system in bipyridine derivatives, where electron delocalization across aromatic rings induces superior resonance stabilization [36].

Post-cycling characterization was performed via CV and ^1^H NMR spectroscopy to elucidate crossover dynamics and redox integrity. As shown in Figure 6b, the post-cycled MV anolyte retained nearly identical redox features compared to pristine MV, demonstrating effective suppression of TEMP-BPy crossover through the cation-selective membrane. In addition, ^1^H NMR analysis (Figure 6c) revealed no detectable proton resonance signals beyond those characteristic of MV. Evidently, these findings confirmed the extremely low crossover of TEMP-BPy, which might bolster the cycling stability of the battery. However, CV characterization of the cycled TEMP-BPy cathode (Figure S12) revealed significant methyl viologen (MV) crossover between redox compartments. This interfacial shuttle phenomenon is postulated to accelerate capacity decay mechanisms, particularly in systems with elevated MV concentrations, where concentration gradient-driven diffusion becomes thermodynamically favorable [11,37].

## 4. Conclusions

In this study, we successfully engineered a 4,4′-bipyridine-ester dual-modified TEMPO derivative (TEMP-BPy) as the catholyte material for neutral AORFBs. TEMP-BPy was synthesized via an efficient two-step protocol involving bromination and quaternization, demonstrating scalability for practical applications. The strong electron-withdrawing bipyridine moieties elevate the redox potential by 60 mV, enabling a theoretical cell voltage of 1.28 V when paired with MV. The enhanced hydrophilicity from pyridine nitrogen coordination causes TEMP-BPy to achieve an aqueous solubility of up to 1.78 M in water solution. The 0.6 M TEMP-BPy/MV AORFB exhibited remarkable cycling stability, demonstrating 99.86% average capacity retention per cycle over 200 cycles and particularly impressive initial stability, with an average capacity retention of 99.997% per cycle over the first 100 cycles, combined with an average Coulombic efficiency of 99.2%. Additionally, it delivered a high energy density of 14.5 Wh L^−1^. The excellent performance of TEMP-BPy/MV AORFB can be attributed to the synergistic effects of the π-conjugated bipyridine framework, which stabilizes radical intermediates, and the enhanced cationic charge density, which effectively suppresses membrane crossover.

## Data Availability

The original contributions presented in this study are included in the article/supplementary material. Further inquiries can be directed to the corresponding author.

## Supplementary Materials

The following supporting information can be downloaded at https://www.mdpi.com/article/10.3390/ma18122770/s1, Synthesis and Characterization of MV. Randles–Ševčík equation for cyclic voltammetry experiments. Scheme S1: Synthesis route of MV. Table S1: Cost analysis of TEMP-BPy. Figure S1: ^1^H NMR spectrum of reduced TEMP-BPy, recorded in D_2_O. TEMP-BPy was reduced by ascorbic acid prior to characterization. ^1^H NMR (400 MHz, D_2_O, δ in ppm): δ 8.88 (s, 2H), 8.66 (s, 2H), 8.34 (s, 2H), 7.85 (s, 2H), 5.21–4.92 (m, 2H), 4.61 (s, 3H), 2.48 (s, 3H), 2.26 (s, 2H), 2.14 (d, J = 14.2 Hz, 2H), 1.78 (s, 2H), and 1.31 (s, 14H). Scheme S2: Synthesis route of TEMP-BPy-TEMP. Figure S2: (a) ESI-MS spectrum of TEMP-BPy. Calculated: 397.2360. Found: 397.2361. (b) ESI-MS full spectrum of the product. TEMP-BPy-TEMP detected at 638.4054. Figure S3: EPR spectrum of TEMP-BPy. Figure S4: FT-IR spectra of TEMP-Br and TEMP-BPy. Table S2: Overview of determined densities and viscosities of TEMP-BPy at various concentrations in 1.5 M NaCl aqueous solution at 20 °C. Figure S5: CV tests of the TEMP-BPy in 1.5 M NaCl aqueous solution. (a) CV plots of the anode part (−0.65 V to −0.2 V) and the cathode part (0.2 V to 0.8 V) with scan rates from 20 to 500 mV s^−1^. (b) Corresponding CV analysis of the redox reaction process of the anode. (c) Corresponding CV analysis of the redox reaction process of the cathode. Figure S6: ^1^H NMR spectrum of MV, recorded in D_2_O. ^1^H NMR (400 MHz, D_2_O, δ in ppm): δ 8.93 (s, 4H), 8.42 (s, 4H), and 4.39 (s, 6H). Figure S7: CV tests of (a) 4-OH-TEMPO (0.2 V to 0.8 V) and (c) MV (−0.8 V to −0.2 V) in 1.5 M NaCl aqueous solution with scan rates varying from 20 to 500 mV s^−1^. (b) Corresponding CV analysis of the redox reaction process of 4-OH-TEMPO. (d) Corresponding CV analysis of the redox reaction process of MV. Figure S8: Charge and discharge voltage plateaus of TEMP-BPy/MV AORFB over 500 cycles at 30 mA cm^−2^. (Each reservoir contained 10 mL of TEMP-BPy and 15 mL of MV, each at a concentration of 0.1 M in a 1.5 M NaCl aqueous solution, with a theoretical storage capacity of 26.8 mAh). Figure S9: CV curves of (a) MV electrolyte after cycling from −0.8 V to −0.2 V and (b) TEMP-BPy electrolyte after cycling from 0.2 V to 0.8 V, scanned for 50 cycles at a scan rate of 50 mV s^−1^. Figure S10: A photograph of the AORFB used. Figure S11: Galvanostatic cycling performance of 0.1 M TEMP-BPy bipolar AORFB system over 80 cycles at 30 mA cm^−2^. (Each reservoir contained 10 mL of 0.1 M TEMP-BPy and 15 mL of 0.1 M TEMP-BPy in a 1.5 M NaCl aqueous solution, with a theoretical storage capacity of 26.8 mAh). Figure S12: CV tests of TEMP-BPy before (black) and after (red) cycling at a scan rate of 50 mV s^−1^. Table S3: A summary of different TEMPO derivatives in the AORFB system.

## Abbreviations

The following abbreviations are used in this manuscript:

AORFB	Aqueous organic redox flow battery
CV	Cyclic voltammetry
LSV	Linear sweep voltammetry
RDE	Rotating disk electrode measurements
TEMPO	2,2,6,6-tetramethylpiperidin-1-oxyl
MV	Methyl viologen
CE	Coulombic efficiency
D	Diffusion coefficient
K_0_	Electron-transfer rate constant
DCM	Dichloromethane
ACN	Acetonitrile
TEMP-BPy	(2,2,6,6-tetramethyl-1-piperidinyloxy)carbonyl-ethyl-(4-(pyridin-4-yl)benzyl) ammonium bromide
