# Supplementary material for: A Bipyridine-Ester Dual-Modified 2,2,6,6-Tetramethylpiperidin-1-oxyl Derivative for Aqueous Organic Redox Flow Batteries
Source: Materials (Basel). 2025 Jun 12;18(12):2770. doi: 10.3390/ma18122770 (PMC12195010; doi:10.3390/ma18122770)
Supplement: Supplementary file 1 [file materials-18-02770-s001.zip › materials-3668205-supplementary.pdf]

A Bipyridine-ester Dual-Modified 2,2,6,6-Tetramethylpiperidin-1-oxyl Derivative for Aqueous Organic Redox Flow Batteries

Qianqian Zheng [1], Yanwen Ren [1], Cuicui He [1], Jingjing Nie [2], and Binyang Du [1,*]

[1] State Key·Laboratory (SKL) of Biobased Transportation Fuel Technology, Department of Polymer Science & Engineering, Zhejiang University, Hangzhou, 310058, China; 22229025@zju.edu.cn (Q.Z.);ywren@zju.edu.cn (Y.R.); 22429001@zju.edu.cn (C.H.)

[2] Department of Chemistry, Zhejiang University, Hangzhou-310058, China; niejj@zju.edu.cn (J.N.)

[*] Correspondence: duby@zju.edu.cn (B.D.)

S1. Experimental section

S1.1 Synthesis and Characterization of MV.

Methyl viologen (MV) was prepared according to the synthetic methods reported in the literature [1], as shown in **Error! Reference source not found.**. In a 250 mL round-bottom flask, 4,4'-bipyridine (7.85 g, 50 mmol) and chloroacetic acid (12.56 g, 135 mmol) were added successively and dissolved in 150 mL of N, N'-dimethylformamide (DMF). The mixture was heated to 135 °C and refluxed for 24 h. After the reaction was completed, it was cooled to room temperature, and the filtrate was removed by suction filtration. The obtained gray precipitate was washed three times successively with hot DMF, chloroform, and dichloromethane. A grayish-white solid MV powder was obtained (yield: 99%). The ^{1}H NMR spectrum confirmed the purity and structure of MV (Figure S5).

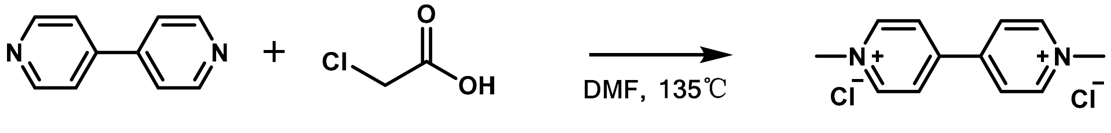

Scheme S1. Synthesis route of MV.

S1.2 Randles - Ševčík equation for cyclic voltammetry experiments.

The Randles - Ševčík equation is an important equation in electrochemistry. It is used to describe the relationship between the peak current and various parameters in a cyclic voltammetry experiment. Mathematically, the Randles–Ševčík equation is expressed as

$$i_p = 2.69 \times 10^5 n^{3/2} A D^{1/2} v^{1/2} C$$

where i_p is the peak current, n is the number of electrons transferred in the redox reaction, A is the area of the electrode surface, D is the diffusion coefficient of the electroactive species, v is the scan rate of the potential, and C is the concentration of the electroactive species.

This equation provides a theoretical basis for quantitative analysis in cyclic voltammetry. By measuring the peak current at different scan rates, we can preliminarily analyze the substance's ability in the redox process [2].

S2. Supplemental tables and figures

The cost of TEMP-BPy and $VOSO_4$ is calculated according to the raw material prices and the yield for the two steps. The cost of the final product is 15.4 yuan/kg. The yield of the material $VOSO_4$ for VRFB developed from V_2O_5 is around 98.6%[3]. Based on this, the commercial VRFBs are 57.2 yuan/kg if the same calculation method is used.

Table S1. Cost analysis of TEMP-BPy.

	Materials	Price[a] (yuan/kg)
Raw	4-OH-TEMPO	10.0
	4-bromobutyryl chloride	16.0
	4,4′-bipyridine	16.0
Product	TEMP-BPy	15.4
Raw	V_2O_5	100
	H_2SO_4	0.55
Product	V-ion ($VOSO_4$)	57.2

a: Prices are sourced from https://b2b.baidu.com/.

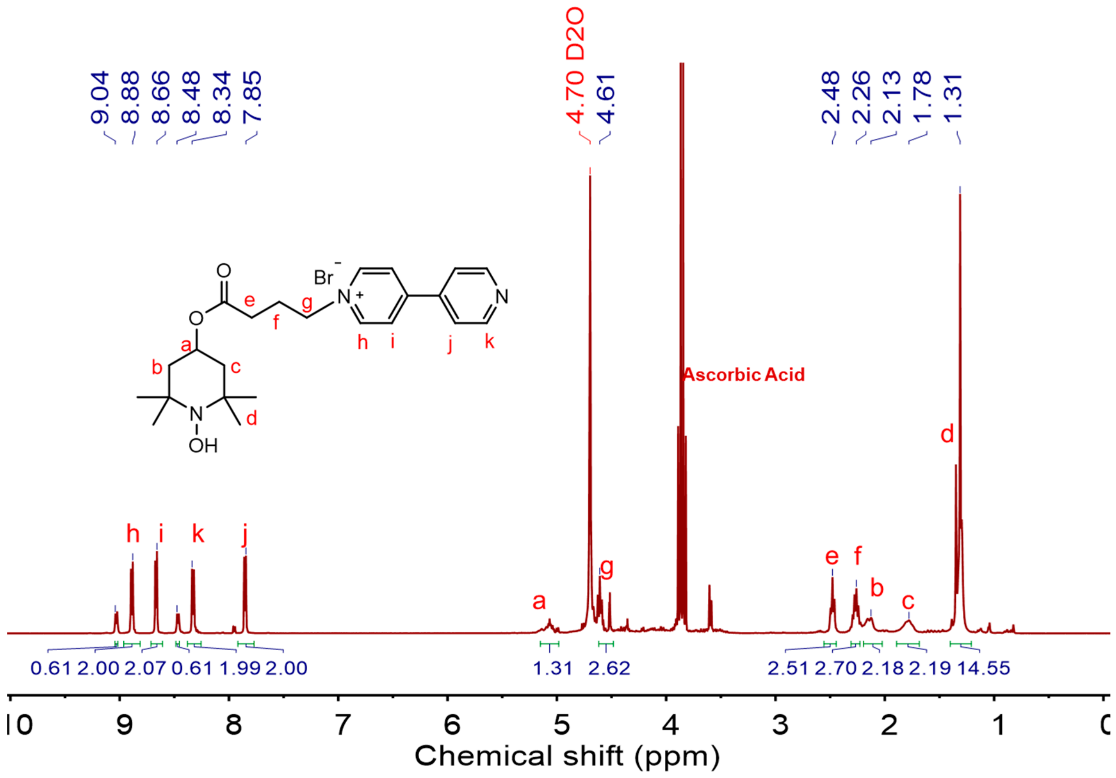

Figure S1. ^1H NMR spectrum of reduced TEMP-BPy, recorded in D_2O. TEMP-BPy was reduced by ascorbic acid prior to characterization. ^1H NMR (400 MHz, D2O, δ in ppm): δ 8.88 (s, 2H), 8.66 (s, 2H), 8.34 (s, 2H), 7.85 (s, 2H), 5.21 – 4.92 (m, 2H), 4.61 (s, 3H), 2.48 (s, 3H), 2.26 (s, 2H), 2.14 (d, J = 14.2 Hz, 2H), 1.78 (s, 2H), and 1.31 (s, 14H).

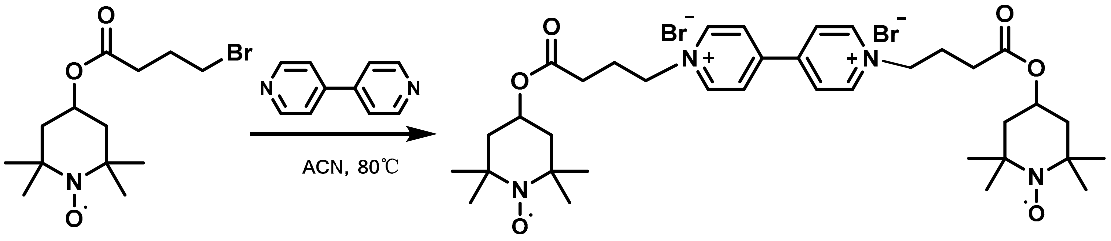

Scheme S2. Synthesis route of by-product TEMP-BPy-TEMP.

In the ^1H NMR, the peaks at δ 9.04 and 8.48 ppm are assigned to the bipyridine structure's 4H of TEMP-BPy-TEMP, and integration shows it accounts for ~15% of the molecule.

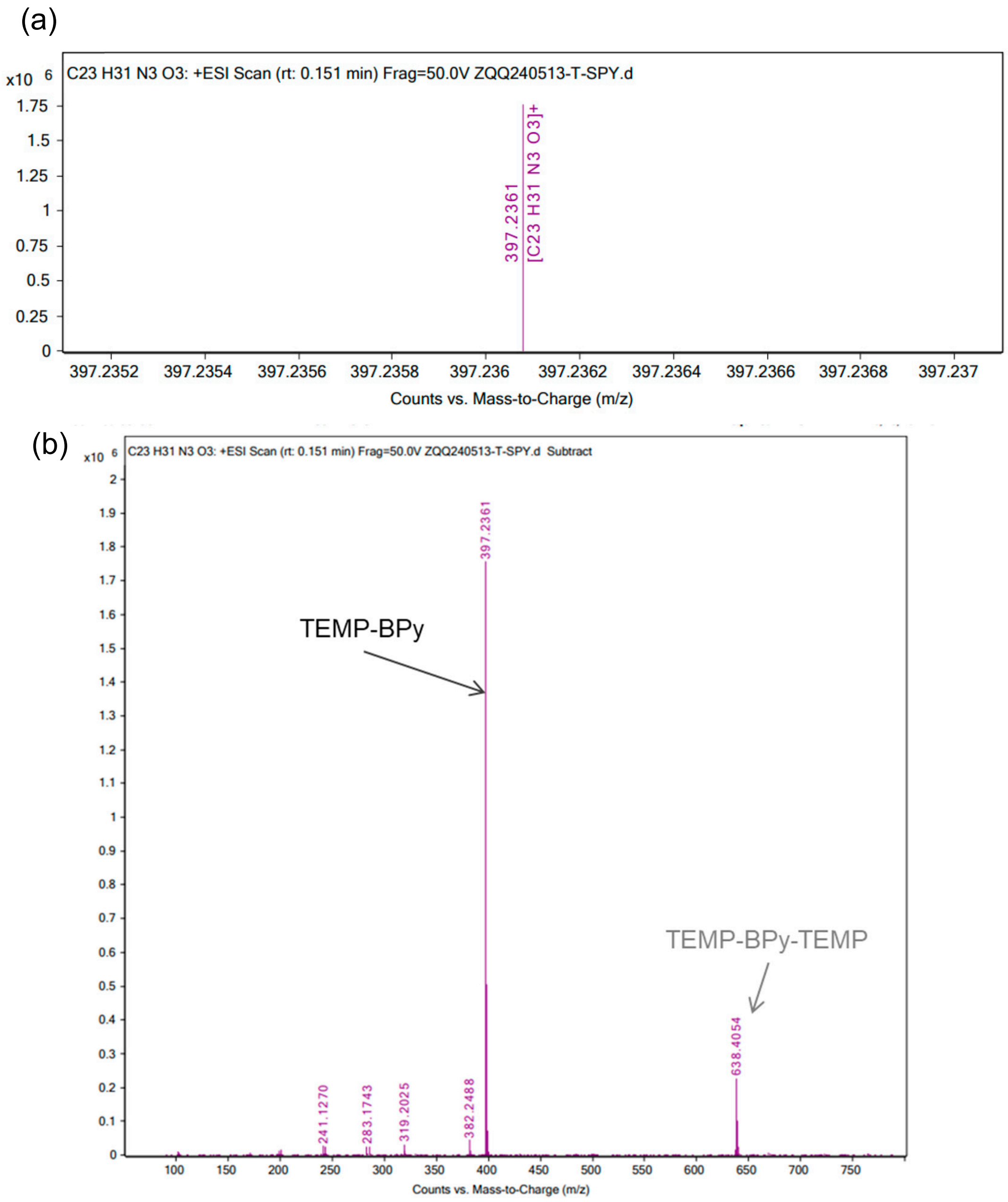

Figure S2. (a) ESI-MS spectrum of TEMP-BPy. Calculated: 397.2360. Found: 397.2361. (b) ESI-MS full spectrum of the product. TEMP-BPy-TEMP detected at 638.4054.

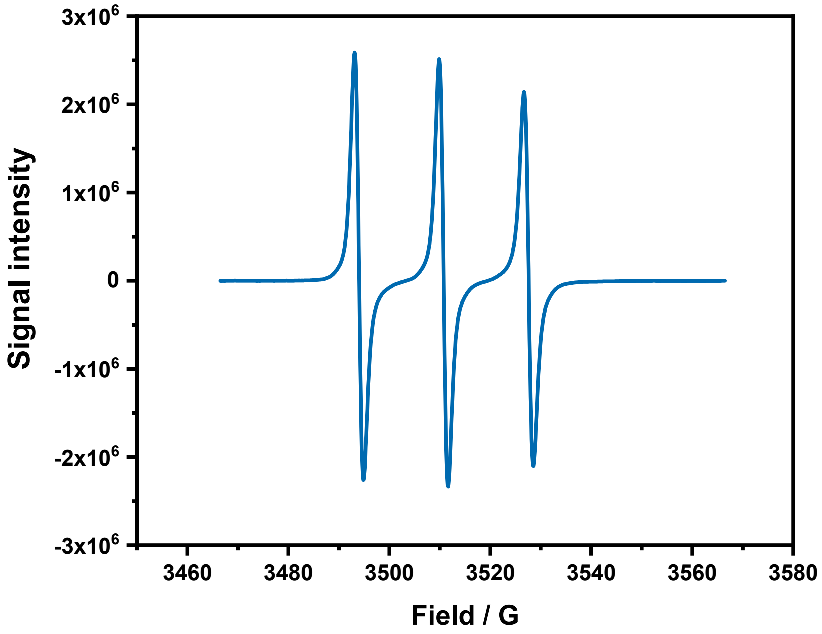

Figure S3. EPR spectrum of TEMP-BPy.

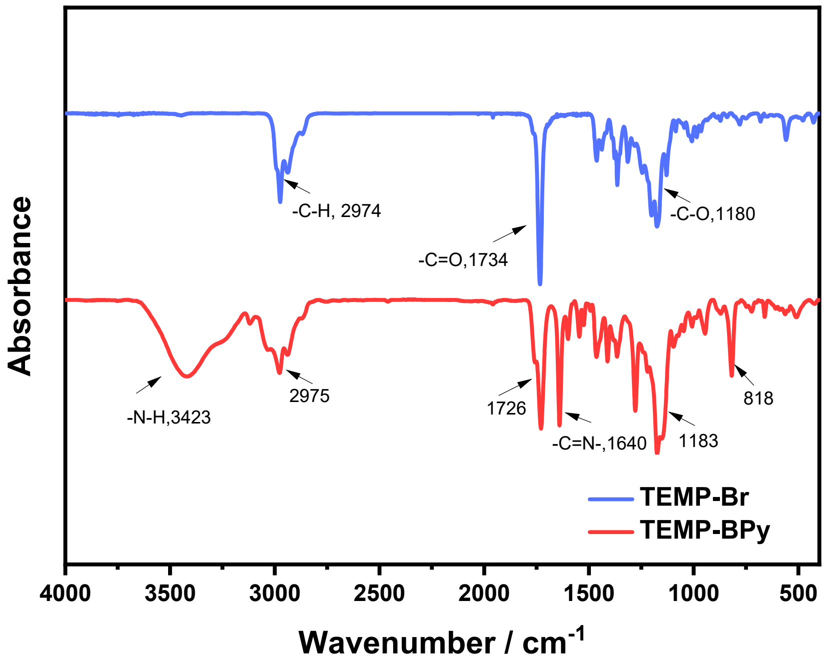

Figure S4. FT-IR spectra of TEMP-Br and TEMP-BPy.

Table S2. Overview of determined densities and viscosities of **TEMP-BPy** at various concentrations in 1.5 M NaCl aqueous solution at 20 °C.

Concentration [mol L^{-1}]	Density [g cm^{-3}]	Drop time of the ball[s]	Dynamic viscosity [mPa s]	Average viscosity [mPa s]
		17.665	1.278	
0.1	1.043	17.627	1.275	1.276
		17.646	1.276	
		18.566	1.341	
0.2	1.053	18.608	1.344	1.342
		18.586	1.3423	
		22.087	1.594	
0.3	1.060	21.848	1.576	1.585
		21.948	1.584	
		26.903	1.940	
0.4	1.063	26.892	1.939	1.940
		26.899	1.940	
		31.653	2.280	
0.5	1.071	31.579	2.274	2.277
		31.624	2.278	
		36.021	2.592	
0.6	1.078	36.087	2.597	2.592
		35.969	2.588	

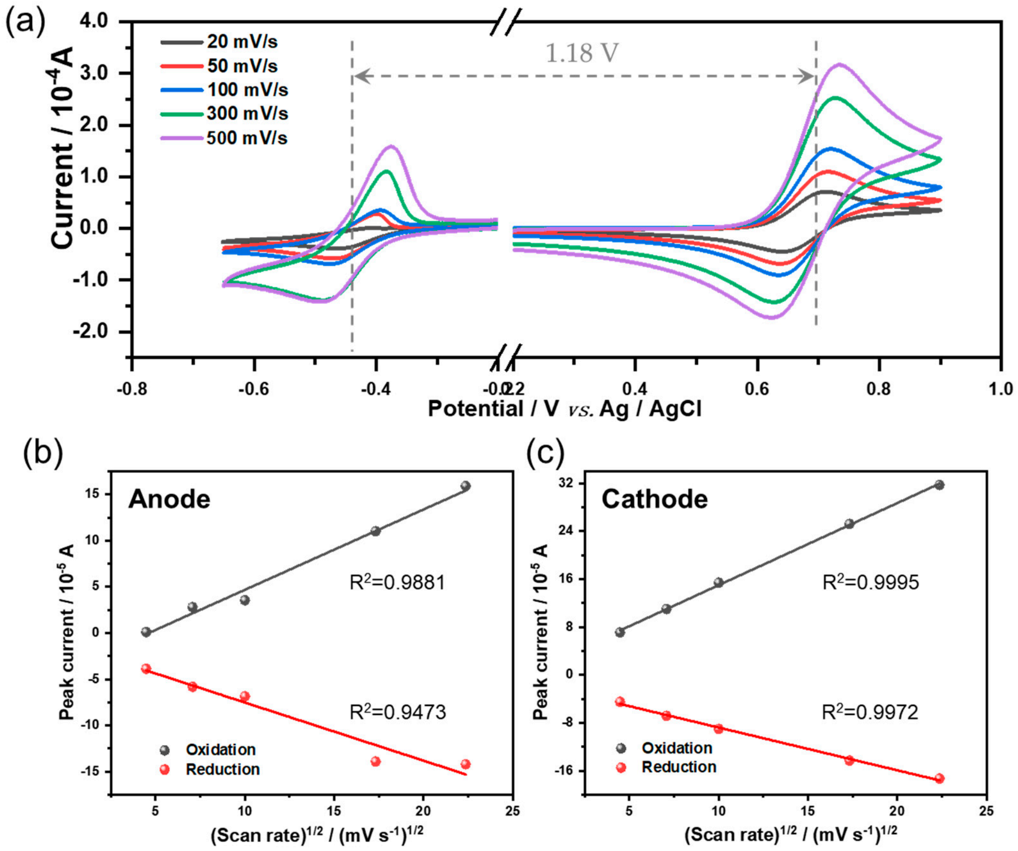

Figure S5. CV tests of the TEMP-BPy in 1.5 M NaCl aqueous solution. (a) CV plots of the anode part (-0.65 V to -0.2 V) and the cathode part (0.2 V to 0.8 V), with scan rates from 20 to 500 mV s^{-1}. (b) Corresponding CV analysis of the redox reaction process of the anode part. (c) Corresponding CV analysis of the redox reaction process of the cathode part.

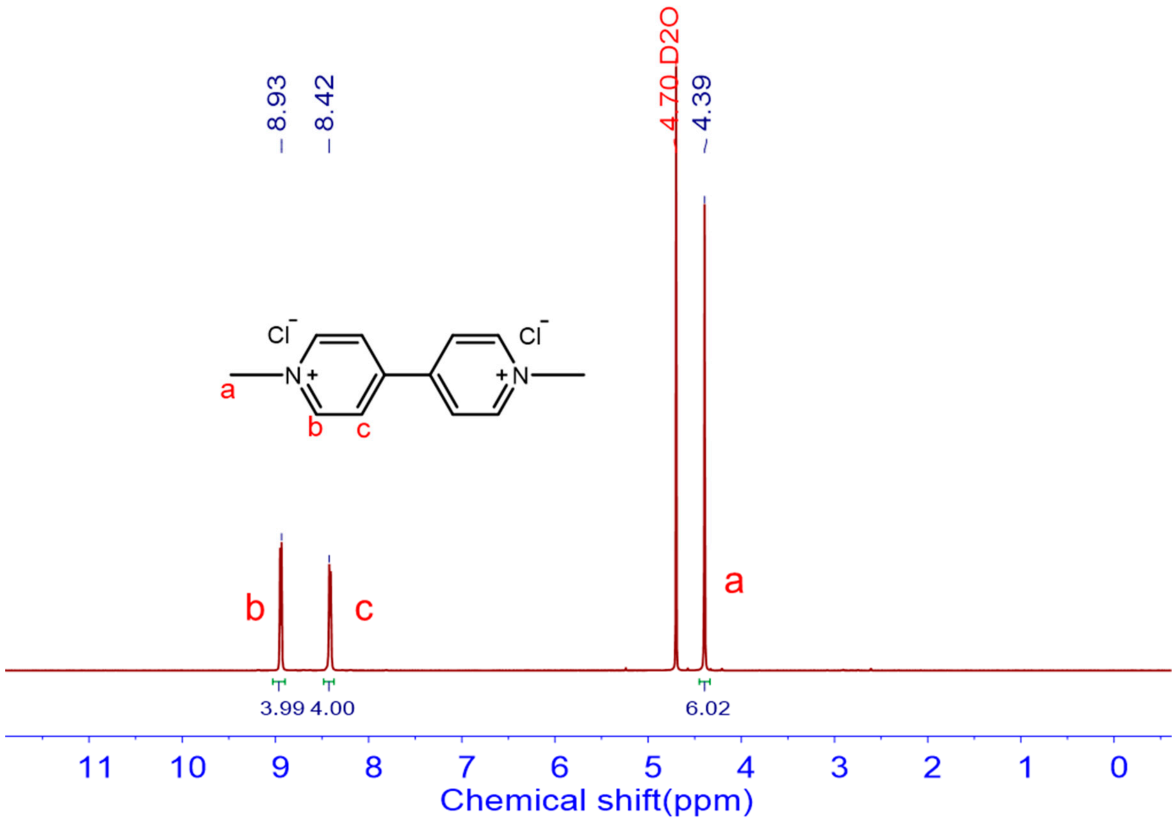

Figure S6. ^{1}H NMR spectrum of MV, recorded in D_2O. ^{1}H NMR (400 MHz, D_2O, δ in ppm): δ 8.93 (s, 4H), 8.42 (s, 4H), and 4.39 (s, 6H).

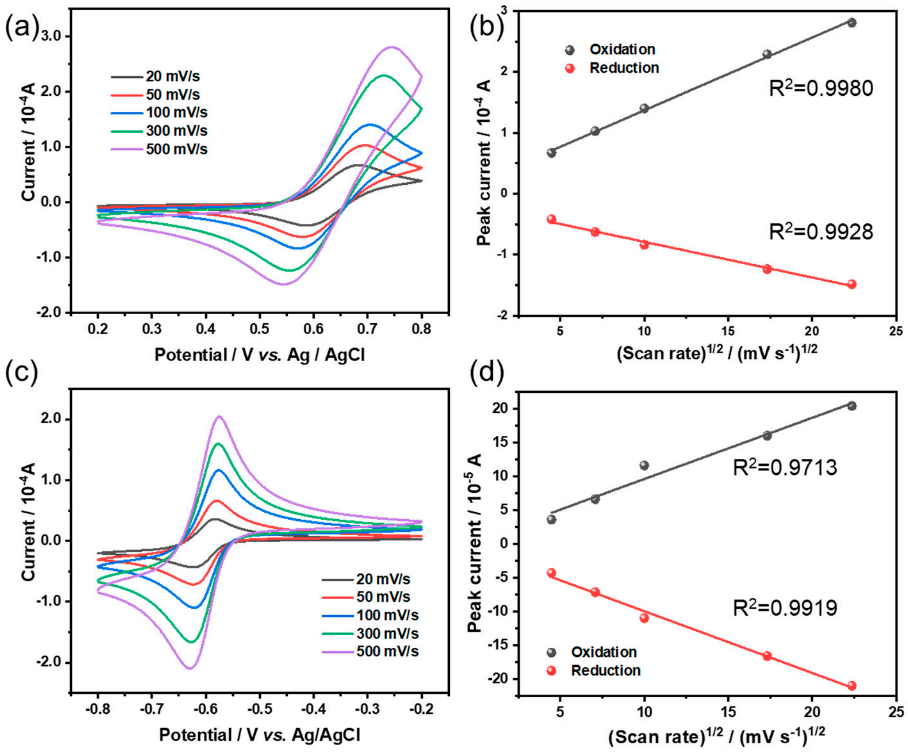

Figure S7. CV tests of the (a) 4-OH-TEMPO (0.2 V to 0.8 V) and (c) MV (-0.8 V to -0.2 V) in 1.5 M NaCl aqueous solution, with scan rates varying from 20 to 500 mV s^{-1}. (b) Corresponding CV analysis of the redox reaction process of 4-OH-TEMPO. (d) Corresponding CV analysis of the redox reaction process of MV.

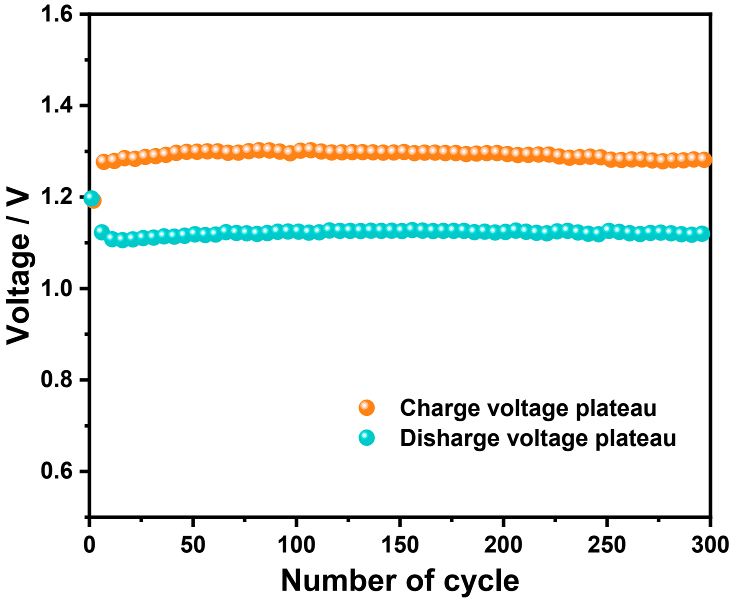

Figure S8. Charge and discharge voltage plateau of TEMP-BPy/MV AORFB over 500 cycles at 30 mA cm^{-2}. (Each reservoir contained 10 mL of TEMP-BPy and 15 mL of MV, each at a concentration of 0.1 M in a 1.5 M NaCl aqueous solution, with a theoretical storage capacity of 26.8 mAh.)

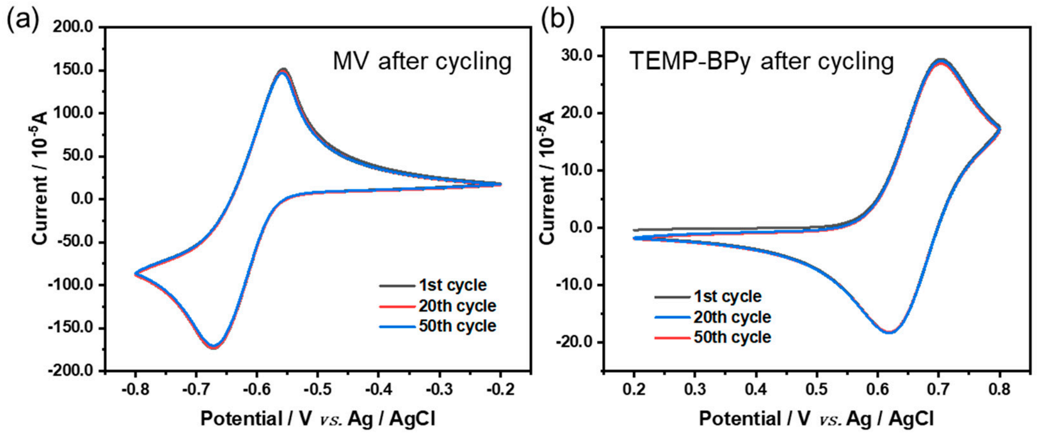

Figure S9. CV curves of (a) MV electrolyte after 500 cycles from -0.8 V to -0.2 V and (b) TEMP-BPy electrolyte after 500 cycles from 0.2 V to 0.8 V, scanned for 50 cycles at a scan rate of 50 mV s^{-1}.

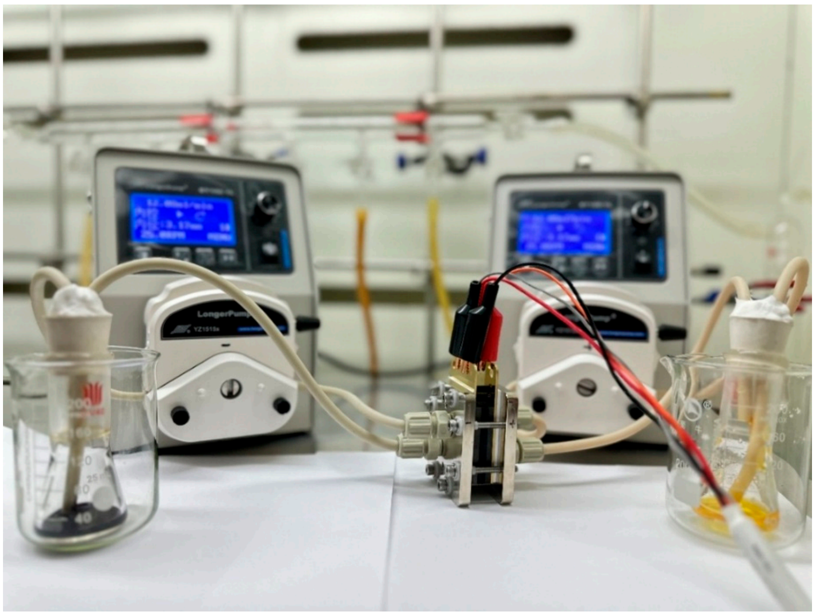

Figure S10. A photograph of the AORFB used.

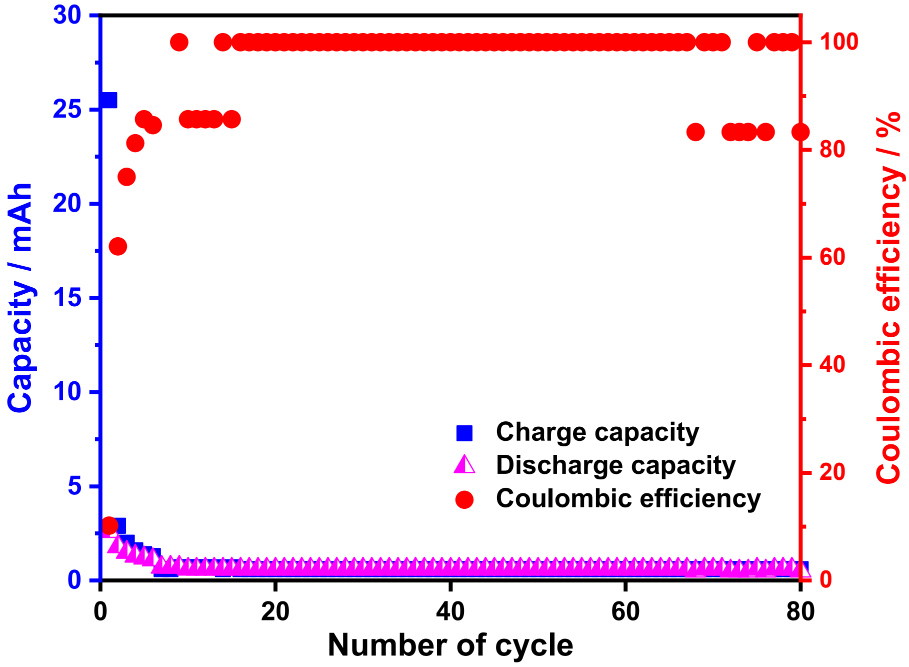

Figure S11. Galvanostatic cycling performance of 0.1 M TEMP-BPy bipolar AORFB system over 80 cycles at 30 mA cm^{-2}. (Each reservoir contained 10 mL of 0.1 M TEMP-BPy and 15 mL of 0.1 M TEMP-BPy in a 1.5 M NaCl aqueous solution, with a theoretical storage capacity of 26.8 mAh.)

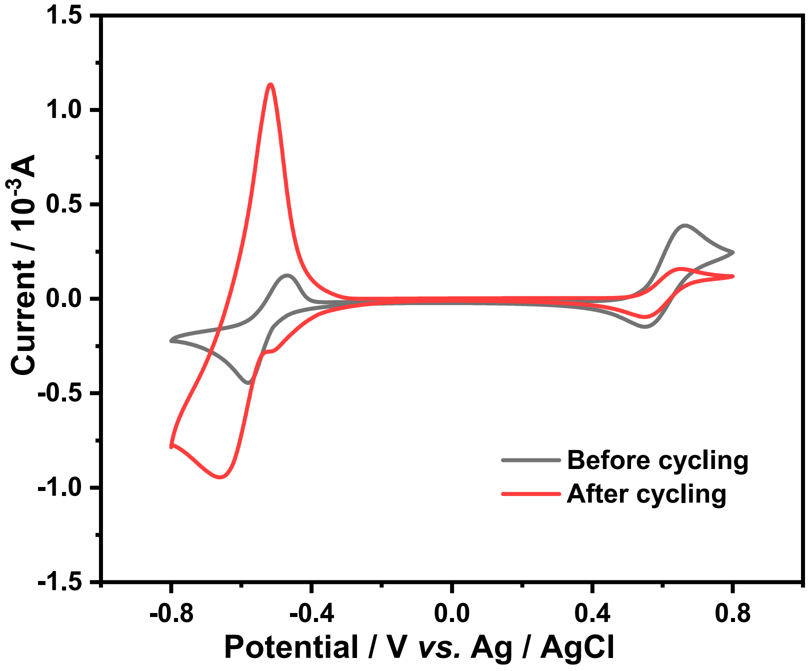

Figure S12. CV tests of TEMP-BPy before (black) and after (red) cycling at a scan rate of 50 mV s^{-1}.

Table S3. A summary of different TEMPO derivatives in the AORFB system.

Cathode /Anode	Chemical structure of cathode	Solubility [mol L^{-1}]	Operated concentration [mol L^{-1}]	Cell Voltage [V]	Material utilization [%]	Energy Density [Wh L^{-1}]	Average capacity fade rate [per cycle]/cycles	Ref
TEMP-BPy /MV	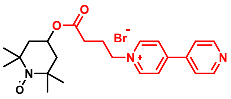	1.78	0.6	1.28	71.1%	14.5	0.003%/100 cycles; 0.14%/200 cycles	This work
TEMPTMA /MV	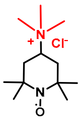	>3.2	2	1.4	<83%	38	0.04%/100 cycles	[4]
TMAP-TEMPO/BTMAP-Vi	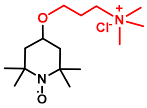	4.6	0.5	1.19	92.4%	14.7	0.025%/200 cycles	[5]
N_2-TEMPO/ BTMAP-Vi	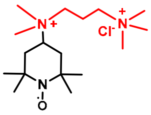	3	0.5	1.35	67.3%	9.05	0.02%/400 cycles	[6]
(TPABPy)Cl_3/ BTMAP-Vi	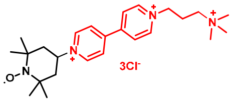	1.76	1.5	1.29	/	19	0.02%/100 cycles	[7]
4-CO_2Na-TEMPO/ $(SPr)_2$V	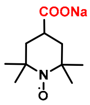	2.5	0.4	1.19	89%	8.95	0.2%/50 cycles	[8]
TMAAcNH-TEMPO /(NPr)2V	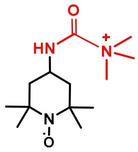	4.3	0.5	1.22	92.6%	16.4	0.014%/1000 cycles	[9]
Pyr-TEMPO / (PyrPV)Cl_4	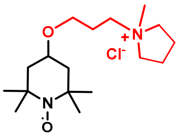	>3.35	0.2	1.57	87%	16.8	0.05%/1000 cycles	[10]

S3. References

1. Fu, H.; Zhang, C.; Wang, H.; Du, B.; Nie, J.; Xu, J.; Chen, L. Stable aqueous redox flow battery assembled in air atmosphere employing an anionic terpolymer as active cathode material. *Journal of Power Sources* **2022**, *545*, 231905, doi:https://doi.org/10.1016/j.jpowsour.2022.231905.
2. González-Meza, O.A.; Larios-Durán, E.R.; Gutiérrez-Becerra, A.; Casillas, N.; Escalante, J.I.; Bárcena-Soto, M. Development of a Randles-Ševčík-like equation to predict the peak current of cyclic voltammetry for solid metal hexacyanoferrates. *Journal of Solid State Electrochemistry* **2019**, *23*, 3123-3133, doi:10.1007/s10008-019-04410-6.
3. Li, L.; Kim, S.; Wang, W.; Vijayakumar, M.; Nie, Z.; Chen, B.; Zhang, J.; Xia, G.; Hu, J.; Graff, G.; et al. A Stable Vanadium Redox-Flow Battery with High Energy Density for Large-Scale Energy Storage. *Advanced Energy Materials* **2011**, *1*, 394-400, doi:https://doi.org/10.1002/aenm.201100008.
4. Janoschka, T.; Martin, N.; Hager, M.D.; Schubert, U.S. An Aqueous Redox - Flow Battery with High Capacity and Power: The TEMPTMA/MV System. *Angewandte Chemie International Edition* **2016**, *55*, 14427-14430, doi:10.1002/anie.201606472.
5. Liu, Y.; Goulet, M.-A.; Tong, L.; Liu, Y.; Ji, Y.; Wu, L.; Gordon, R.G.; Aziz, M.J.; Yang, Z.; Xu, T. A Long-Lifetime All-Organic Aqueous Flow Battery Utilizing TMAP-TEMPO Radical. *Chem* **2019**, *5*, 1861-1870, doi:10.1016/j.chempr.2019.04.021.
6. Hu, B.; Hu, M.; Luo, J.; Liu, T.L. A Stable, Low Permeable TEMPO Catholyte for Aqueous Total Organic Redox Flow Batteries. *Advanced Energy Materials* **2022**, *12*, 2102577, doi:https://doi.org/10.1002/aenm.202102577.
7. Hu, S.; Wang, L.; Yuan, X.; Xiang, Z.; Huang, M.; Luo, P.; Liu, Y.; Fu, Z.; Liang, Z. Viologen-Decorated TEMPO for Neutral Aqueous Organic Redox Flow Batteries. *Energy Material Advances* **2021**, *2021*, doi:doi:10.34133/2021/9795237.
8. Liu, B.; Tang, C.W.; Jiang, H.; Jia, G.; Zhao, T. Carboxyl-Functionalized TEMPO Catholyte Enabling High-Cycling-Stability and High-Energy-Density Aqueous Organic Redox Flow Batteries. *ACS Sustainable Chemistry & Engineering* **2021**, *9*, 6258-6265, doi:10.1021/acssuschemeng.0c08946.
9. Fan, H.; Wu, W.; Ravivarma, M.; Li, H.; Hu, B.; Lei, J.; Feng, Y.; Sun, X.; Song, J.; Liu, T.L. Mitigating Ring - Opening to Develop Stable TEMPO Catholytes for pH - Neutral All - Organic Redox Flow Batteries. *Advanced Functional Materials* **2022**, *32*, doi:10.1002/adfm.202203032.
10. Pan, M.; Gao, L.; Liang, J.; Zhang, P.; Lu, S.; Lu, Y.; Ma, J.; Jin, Z. Reversible Redox Chemistry in Pyrrolidinium - Based TEMPO Radical and Extended Viologen for High - Voltage and Long - Life Aqueous Redox Flow Batteries. *Advanced Energy Materials* **2022**, *12*, doi:10.1002/aenm.202103478.
